# Choroidal Venous Architecture and Polypoidal Lesion Distribution in Subtypes of Polypoidal Choroidal Vasculopathy Using Widefield Imaging

**DOI:** 10.1167/tvst.14.9.7

**Published:** 2025-09-04

**Authors:** Yining Zhang, Miaoling Li, Guiqin He, Yuhong Gan, Xuenan Zhuang, Xuelin Chen, Jiaxin Pu, Yongyue Su, Xinlei Hao, Feng Wen

**Affiliations:** 1 State Key Laboratory of Ophthalmology, Zhongshan Ophthalmic Center, Sun Yat-Sen University, Guangdong Provincial Key Laboratory of Ophthalmology and Visual Science, Guangdong Provincial Clinical Research Center for Ocular Diseases, Guangzhou, China

**Keywords:** polypoidal choroidal vasculopathy, widefield imaging, choroidal vortex veins, choroidal vascular hyperpermeability

## Abstract

**Purpose:**

To investigate the spatial relationship between choroidal vortex veins (VVs), choroidal watershed zones (CWZs), and polypoidal lesion distribution in different subtypes of polypoidal choroidal vasculopathy (PCV) categorized by choroidal vascular hyperpermeability (CVH) status.

**Methods:**

This retrospective study analyzed 58 treatment-naïve PCV eyes using widefield imaging to map dominant VVs, CWZs, and lesion locations. Eyes were stratified into CVH (*n* = 32) and non-CVH (*n* = 26) groups. Comparative analyses were performed to evaluate the differences between PCV eyes and their unaffected fellow eyes, as well as between CVH and non-CVH eyes.

**Results:**

PCV eyes demonstrated significantly increased numbers of dominant VVs (*P* = 0.001) and temporal VV predominance (*P* = 0.039) compared to fellow eyes. Lesion distribution patterns differed significantly between CVH and non-CVH groups (*P* = 0.04). Although 71.8% of lesions (112/156) localized to dominant VV quadrants in both groups, CVH eyes showed greater CWZ involvement (23.9% vs. 10.3% in non-CVH; *P* = 0.029).

**Conclusions:**

The findings indicate that choroidal venous architecture and CVH-driven pathological changes may contribute synergistically to PCV pathogenesis, highlighting the interplay between anatomical vulnerability and pathological processes in PCV development.

**Translational Relevance:**

This study links choroidal VV distribution to PCV lesion localization, enabling risk-stratified imaging protocols. The CVH-specific CWZ involvement further guides personalized monitoring and hemodynamic-targeted adjuvant therapies.

## Introduction

Polypoidal choroidal vasculopathy (PCV) is characterized by two key components that have been newly assigned the nomenclature of “polypoidal lesion” and “branching neovascular network (BNN)” by the Asia-Pacific Ocular Imaging Society (APOIS) PCV Workgroup^1^ and is currently considered a subtype of neovascular age-related macular degeneration (nAMD). However, due to notable differences in histology and clinical characteristics such as hyperpermeability and thickening of the choroid in PCV, there is a continuing discussion on whether PCV belongs in the spectrum of nAMD or pachychoroid diseases.

The pachychoroid disease (PCD) spectrum is considered to be a group of conditions characterized by a thick choroid and retinal pigment epithelial changes, with or without associated retinal abnormalities. As a result, the name “pachychoroid” suggests choroidal congestion and hyperpermeability, which are shown on indocyanine green angiography (ICGA) as dilated choroidal vessels, choroidal vascular hyperpermeability (CVH), and other distinctive features.^2^^,^^3^ CVH, a hallmark of pachychoroidopathy, is observed in 34.7% to 59.3% of PCV patients, which suggests that choroidal vascular remodeling may be involved in PCV pathogenesis.^4^^–^^6^

Compared to PCV patients without CVH, patients with CVH typically have thicker choroid and worse outcomes after intravitreal anti-vascular endothelial growth factor (anti-VEGF) treatment.^7^^–^^11^ This suggests that the pathophysiology of PCV may be influenced by the differences in choroidal vascularity between the two subtypes.^12^^,^^13^ Some pathological studies have confirmed that VEGF expression is negative in certain PCV eyes, and venous stasis can be observed in small veins.^14^^,^^15^ This suggests that the pathogenesis of some PCV cases differs from that of typical AMD and instead resembles certain PCDs, which may associated with vortex vein (VV) congestion and stasis.^2^^,^^15^ With the increasing adoption of widefield (WF) multimodal imaging, emerging image-based evidence underscores the role of choroidal vascular anomalies, particularly VV abnormalities, in disease pathogenesis. Utilizing swept-source optical coherence tomography (SS-OCT), Hiroe and Kishi^16^ identified asymmetric dominant VVs in the posterior pole in a subset of normal subjects (15/39) and in all eyes (38/38) with central serous chorioretinopathy (CSC). Similarly, He et al.^17^ reported dominant VVs in the majority of PCD eyes, excluding PCV (57/68), and Hao et al.^18^ observed dominant VVs in most CSC eyes (125/165). These studies collectively suggest that asymmetric dominant VVs may serve as a predisposing factor for PCD.

However, research on the distribution of dominant VVs in PCV eyes and their spatial relationship with polypoidal lesions remains limited. Furthermore, the differences in dominant VVs and polypoidal lesion distribution between PCV eyes with and without CVH have yet to be investigated. This study leverages WF-ICGA and WF-OCTA to integrate venous anatomy and lesion distribution in order to identify anatomical and pathological factors underlying PCV pathogenesis.

## Methods

### Patients and Study Population

This retrospective observational study was conducted at the Zhongshan Ophthalmic Center from May 2023 to September 2024. The study received approval from the Institutional Review Board of the Zhongshan Ophthalmic Center (2020KYPK063). Written informed consent was obtained from each subject. All research and data collection methods adhered to the tenets of the Declaration of Helsinki.

The study enrolled 58 consecutive patients with newly diagnosed, treatment-naïve PCV who had undergone WF-ICGA and WF-OCTA. The diagnosis of PCV was based on the first visit when WF-ICGA was acquired and confirmed by the identification of polypoidal lesions with BNN observed in ICGA and OCTA. The diagnosis was independently confirmed by two experienced ophthalmologists (YZ and XC), and any discrepancies were resolved through consensus discussion.

The inclusion criteria for the PCV-affected eyes were as follows: (1) PCV eyes confirmed by ICGA; (2) no previous treatment; and (3) refractive errors ranging from −3.0 diopters (D) to +1.5 D. The exclusion criteria included low-quality widefield images, obscured fundus images due to media opacities, massive hemorrhages that obscured detection of the PCV lesion, a history of intraocular surgery or laser interventions including photodynamic therapy, previous ocular trauma, and the presence of other concomitant retinal diseases.

For a subanalysis comparing choroidal vascular features, the corresponding contralateral eyes of the enrolled PCV-affected eyes were assessed for eligibility to serve as an unaffected fellow eye control group. Inclusion in this control group required meeting the following criteria on multimodal imaging: (1) a complete absence of polypoidal lesions, BNN, or any other signs of choroidal neovascularization; (2) no evidence of intraretinal or subretinal fluid or pigment epithelial detachments; and (3) no features of typical AMD or other forms of neovascularization.

**Figure 1.**
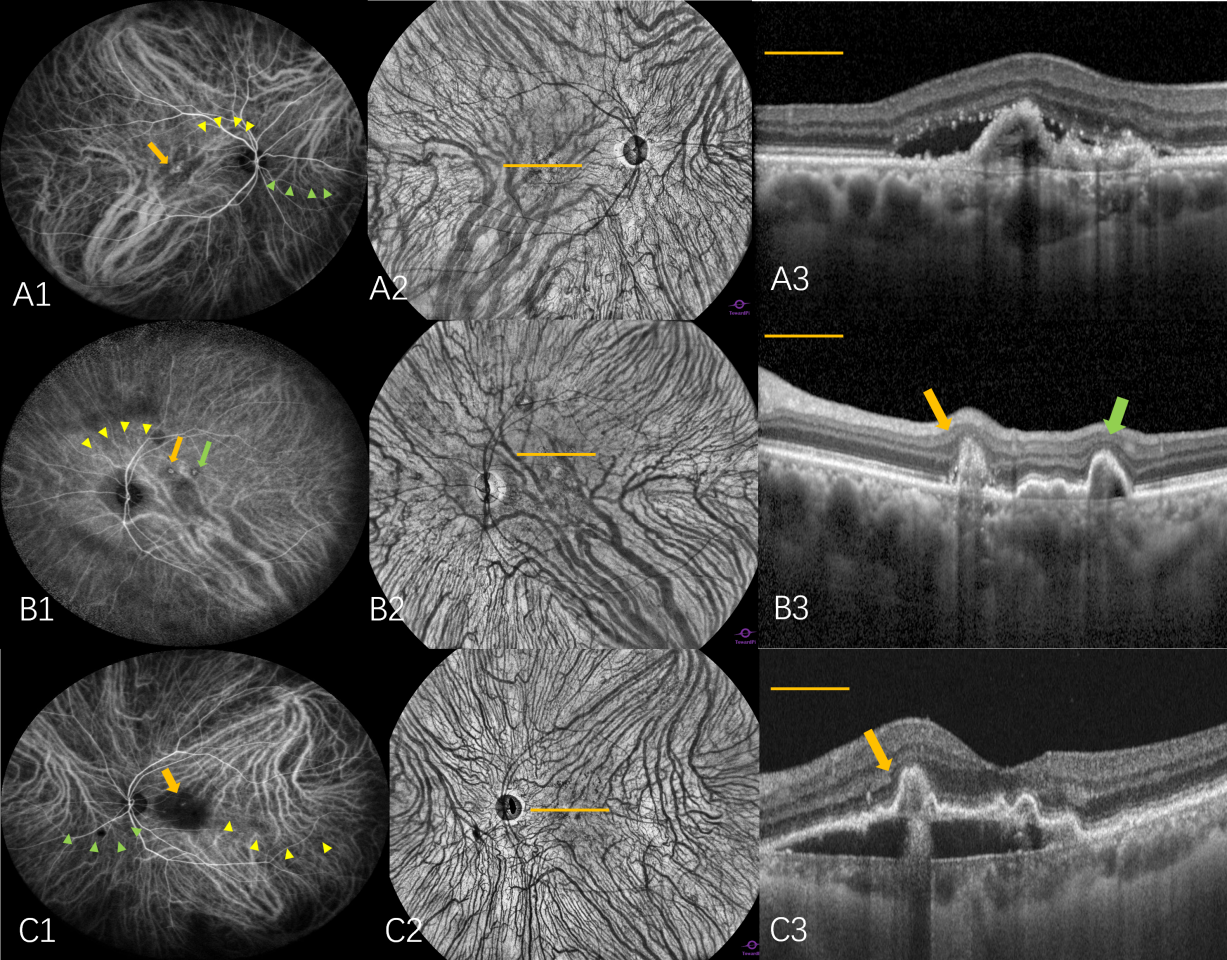
Asymmetric dominant VVs and polypoidal lesions within the dominant VV region visualized using WF-ICGA and en face OCTA. (**A1**–**A3**) Two dominant VVs were observed in the SN and IT regions of the PCV eye. The distal end of the inferotemporal VV (*yellow arrowheads*) crosses the macula, and the terminal of the SN VV (*green arrowheads*) crosses the optic disc. A polypoidal lesion (*orange arrow*) was located within the IT dominant VV area and was classified as type 1 MNV. (**B1**–**B3**) A dominant VV was present in the IT region of the PCV eye, with its distal end (*yellow arrowheads*) crossing the macula. Two polypoidal lesions were distributed within the IT dominant VV area and were classified as being located in the IT quadrant, despite their physical appearance suggesting the ST quadrant. One polypoidal lesion was classified as mixed type 1 and type 2 MNV (*orange arrow*), and another lesion was classified as type 1 MNV (*green arrow*). (**C1**–**C3**) Two dominant VVs were identified in the SN and ST regions of the PCV eye. The distal end of the ST VV (*yellow arrowheads*) crosses the macula, and the terminal of the SN VV (*green arrowheads*) crosses the optic disc. A polypoidal lesion (*orange arrow*) was located within the ST dominant VV area and was classified as being located in the ST quadrant. The polypoidal lesion shown in C3 (*yellow arrow*) was classified as a mixed type 1 and type 2 MNV.

All participants underwent comprehensive ophthalmic examinations, including best-corrected visual acuity testing (BCVA) using the logarithm of minimum angle of resolution (logMAR) and Early Treatment of Diabetic Retinopathy Study (ETDRS) charts, intraocular pressure, slit-lamp microscopy, ophthalmoscopic examination, fluorescein angiography (FA), WF-ICGA, structural OCT (Heidelberg Engineering, Heidelberg, Germany), and WF-OCTA (BM400K; TowardPi Medical Technology Co., Ltd., Beijing, China). The built-in software of the OCTA system was used to automatically calculate the subfoveal choroidal thickness (SFCT), three-dimensional choroidal vascularity index (3D CVI), choroidal vascular volume per area (CVV/a), and choroidal stromal volume per area (CSV/a). The BNN area was manually delineated on the en face avascular slab of the OCTA scan using the built-in freehand drawing tool. For the macular neovascularization (MNV) type, if the polypoidal lesions are mixed with type 2 MNV either at the location of polypoidal lesions or BNN, it would be classified as mixed type 1 and type 2 MNV. If not, it would be classified as type 1 MNV (Fig. 1).

### UWF Image Acquisition and Measurements

WF-ICGA for all PCV eyes was acquired during the early and middle phases, and the duration of ICGA in this study was at least 30 minutes. The session for the main affected eye was videotaped during 15 seconds after choroidal vein developed. In order to observe the full extent of the VVs, WF-ICGA images were obtained centered on the fovea of both eyes for 1 minute, followed by the other eight fixation points: superior, superotemporal (ST), temporal, inferotemporal (IT), inferior, inferonasal (IN), nasal, and superonasal (SN). WF-OCTA was performed for all eyes with the 24-mm × 20-mm scan pattern centered on the fovea with a wavelength centered at 1060 nm and a scan rate of 400,000 A-scans per second.

**Figure 2.**
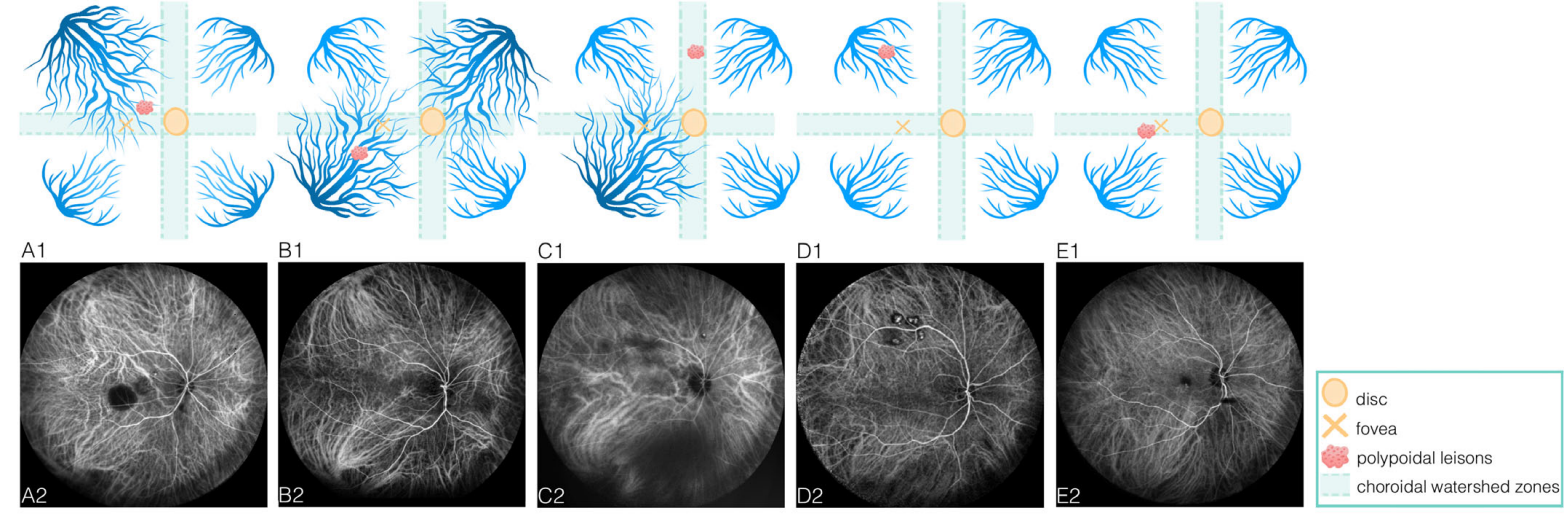
Illustrations of Choroidal Vein Drainage Patterns and Polypoidal Lesion Locations. (**A1**, **A2**) A dominant vortex vein (VV) is observed in the superotemporal (ST) quadrant, with its distal end extending across the macular fovea. A polypoidal lesion is situated within the region of the dominant ST VV. (**B1**, **B2**) Two dominant VVs are identified in the inferotemporal (IT) and superonasal (SN) quadrants of the PCV eye. The terminal of the IT VV traverses the macular fovea, while the terminal of the SN VV crosses the optic disc center. The polypoidal lesion is located within the region of the dominant IT VV. (**C1**, **C2**) A dominant VV is detected in the inferotemporal (IT) quadrant of the PCV eye, with its distal end crossing the macular fovea. A polypoidal lesion is positioned within the vertical choroidal watershed zone (CWZ). (**D1**, **D2**) Symmetric distribution of the choroidal venous drainage pathway reveals both horizontal and vertical CWZs. The horizontal CWZ passes through the macular region and optic disc, while the vertical CWZ traverses the papilla. Polypoidal lesions are located in the superotemporal (ST) quadrant based on their anatomical position. (**E1**, **E2**) Symmetric distribution of the choroidal venous drainage pathway highlights the horizontal and vertical CWZs. The polypoidal lesion is situated within the horizontal CWZ.

**Figure 3.**
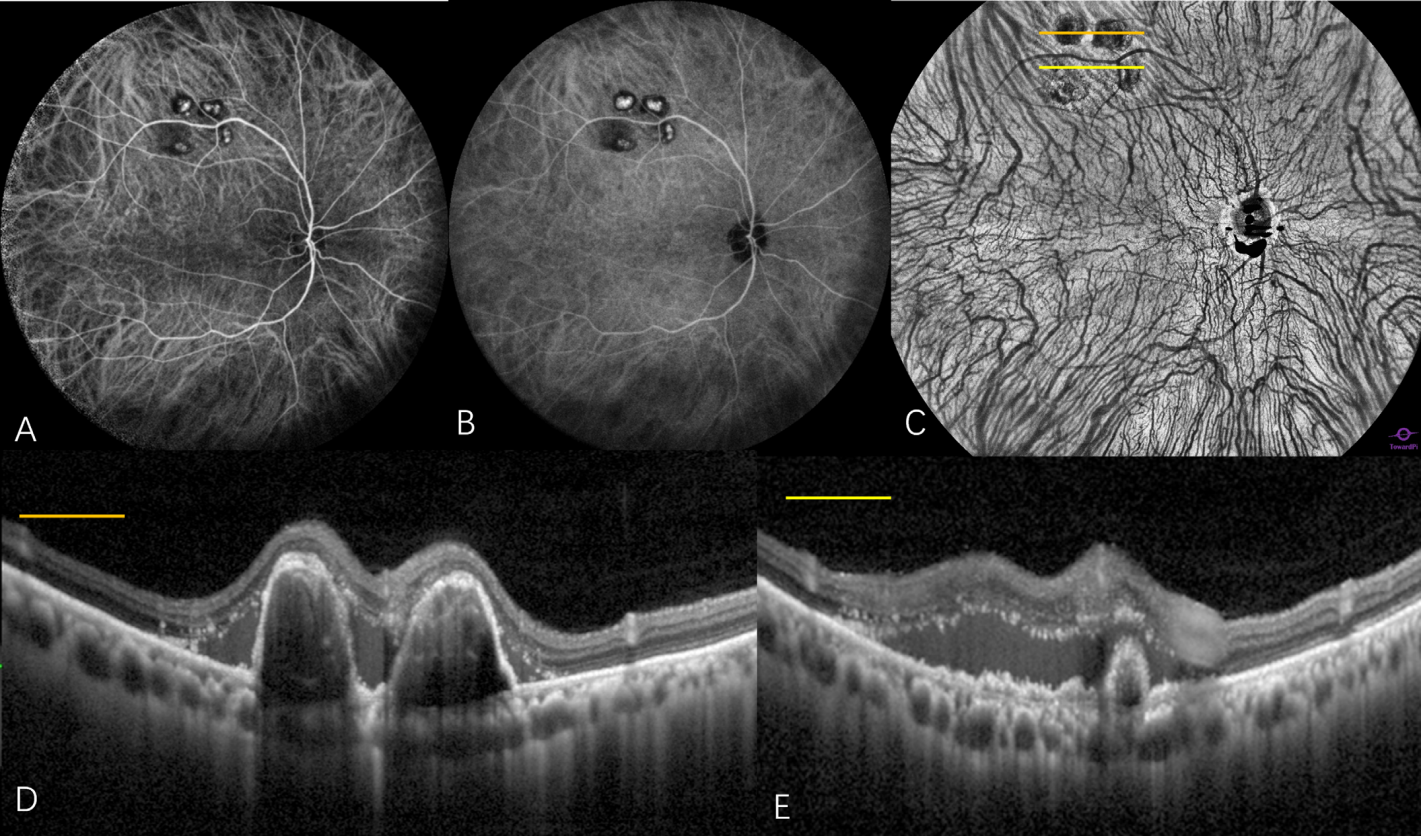
Multimodal widefield imaging features of a PCV case exhibiting symmetrical choroidal venous architecture without CVH. (**A**) Early-phase ICGA (53 seconds) revealed well-demarcated horizontal and vertical choroidal watershed zones without evidence of choroidal venous dilation or engorgement. (**B**) Mid-phase ICGA (7 minutes, 53 seconds) demonstrated progressive fading of watershed zones with absence of CVH. (**C**) En face WF-OCTA illustrates symmetrical branching patterns of choroidal veins with polypoidal lesions localized to the ST quadrant. (**D**, **E**) Corresponding cross-sectional OCT and B-scans confirmed the presence of characteristic polypoidal structures exhibiting typical dome-shaped protrusions from the choroid.

**Figure 4.**
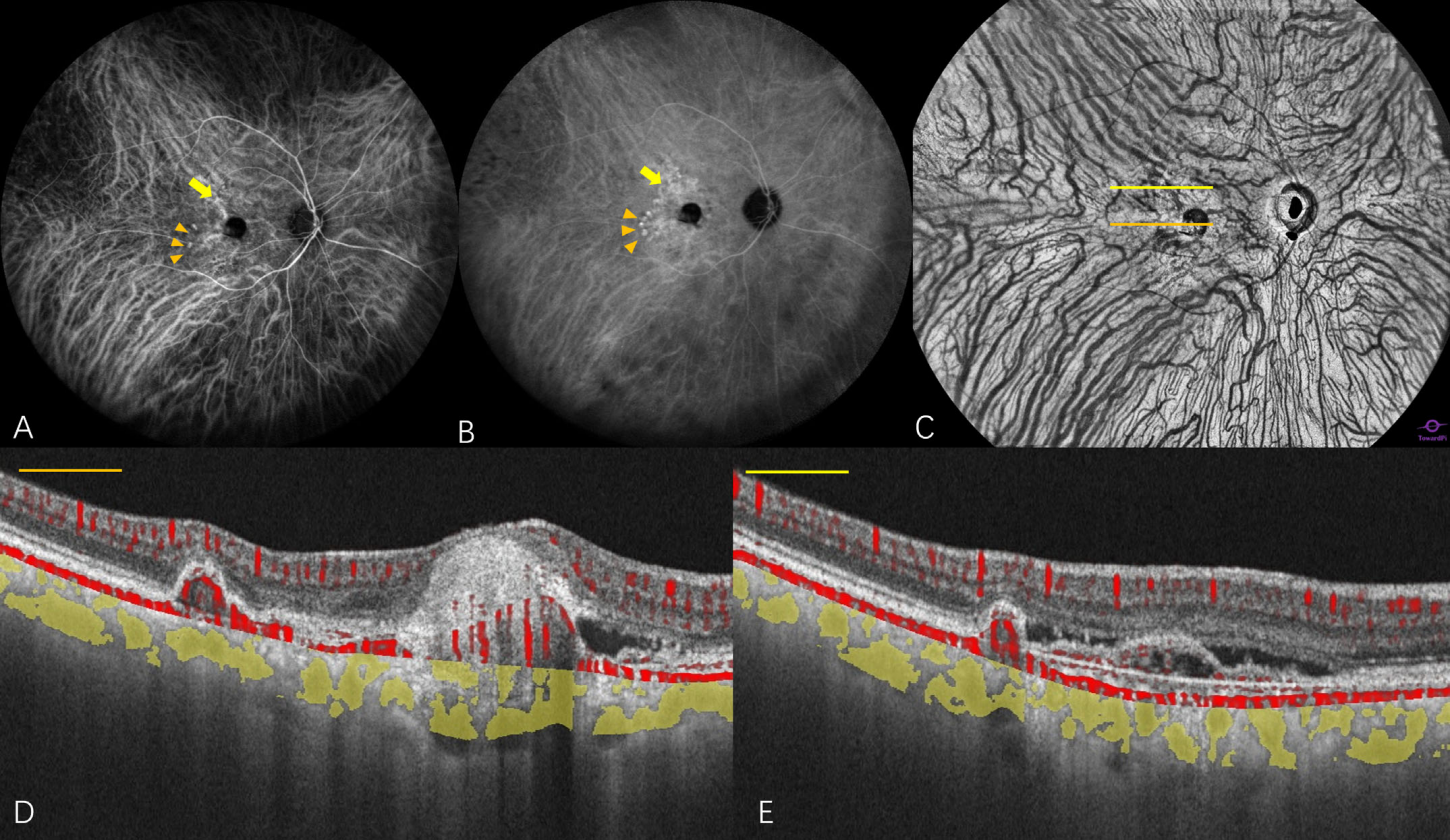
Multimodal widefield imaging features of a PCV case exhibiting symmetrical choroidal venous architecture with CVH. (**A**) Early-phase ICGA (35 seconds) demonstrated distinct horizontal and vertical CWZs. No expanded or engorged choroidal veins were observed. (**B**) Mid-phase ICGA (7 minutes, 53 seconds) revealed CVH. Polypoidal lesions are visible within the CWZ (*orange arrowhead*) and the ST quadrant (*yellow arrow*), as identified based on their anatomical positions in both early and mid-phase ICGA. (**C**) En face WF-OCTA illustrates the symmetric distribution of choroidal veins, with polypoidal lesions localized within the CWZ (*orange line*) and ST quadrant (*yellow line*). (**D**, **E**) Cross-sectional OCTA B-scans depict the polypoidal lesions within the CWZ (*orange line*) and ST quadrant (*yellow line*).

Hayreh^19^^,^^20^ demonstrated that choroidal venous drainage routes are categorized into four distinct quadrants (ST, IT, SN, and IN) based on physiological horizontal and vertical choroidal watershed zones (CWZs). A CWZ was evaluated in ICGA, which is a transient hypofluorescent area that develops and then quickly vanishes in the early phase of ICGA.^21^^,^^22^ Variations of the watershed zones have been identified, with the horizontal watershed zone typically recognized as traversing the macular area and the vertical watershed zone extending across the optic disc. Thus, if the distal end of the VV crossed the macular fovea or optic disc center, it was defined as a dominant VV in our study.^2^^,^^17^^,^^18^ The number and location of dominant VVs were identified using WF-ICGA images and en face WF-OCTA images, and the distribution of choroidal drainage channels was asymmetric when a dominant VV was present but symmetric when it was not. Correspondingly, the number and location of polypoidal lesions of PCV eyes were recorded. When polypoidal lesions were found within dominant VVs area, they were classified into the quadrant corresponding to the dominant VV (Figs. 1, 2A, 2B). Those not situated within dominant VV areas or without dominant VVs were categorized based on their anatomical position into either the relevant quadrant or CWZ (Figs. 2C–E, 3, 4).

**Figure 5.**
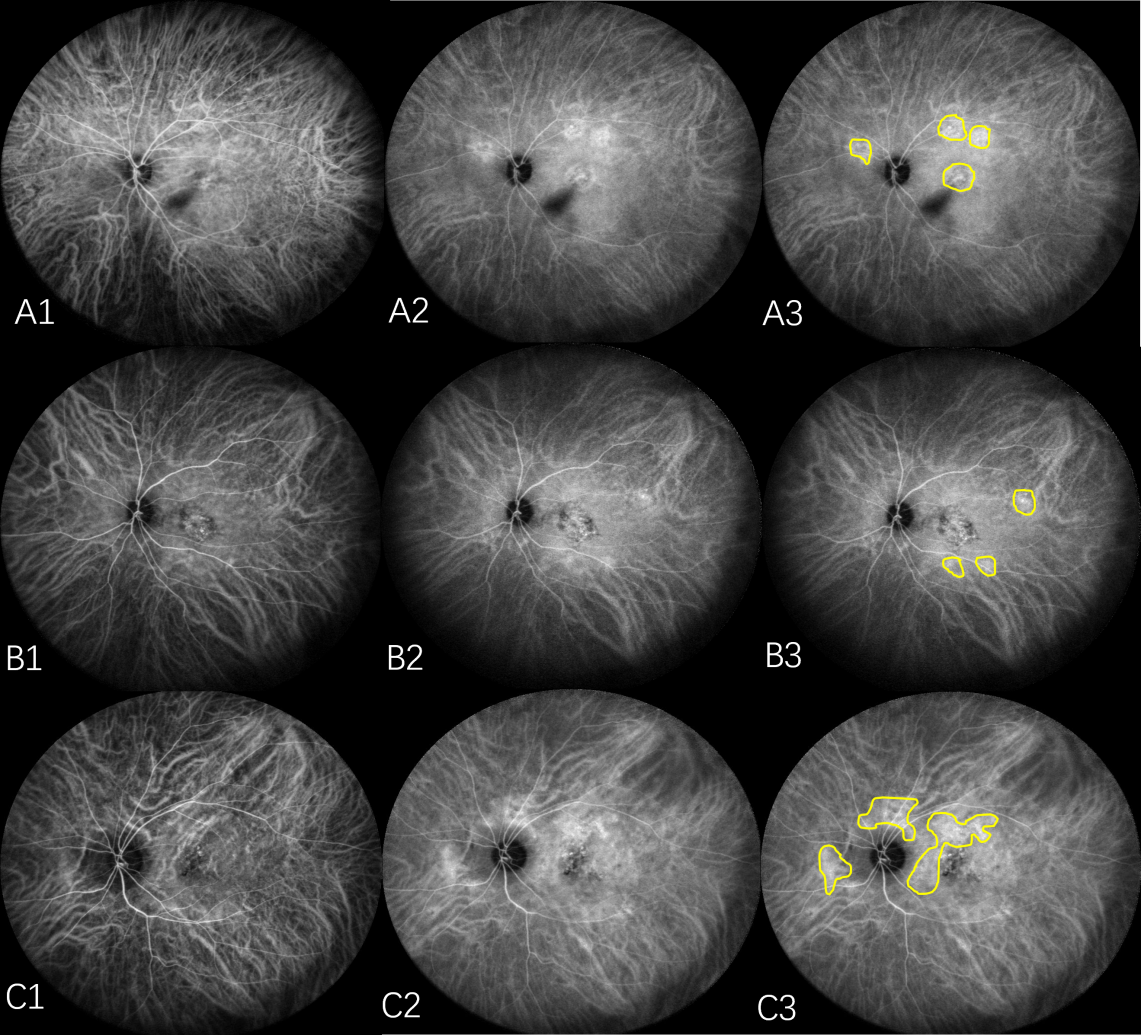
Representative cases of PCV with CVH. (**A1**, **B1**, **C1**) Early-phase ICGA revealed dilation of choroidal veins. (**A2**, **B2**, **C2**) Late-phase ICGA showed the CVH area with multifocal hyperfluorescence. (**A3**, **B3**, **C3**) CVH areas with multifocal hyperfluorescence were demarcated by a *yellow outline*.

**Figure 6.**
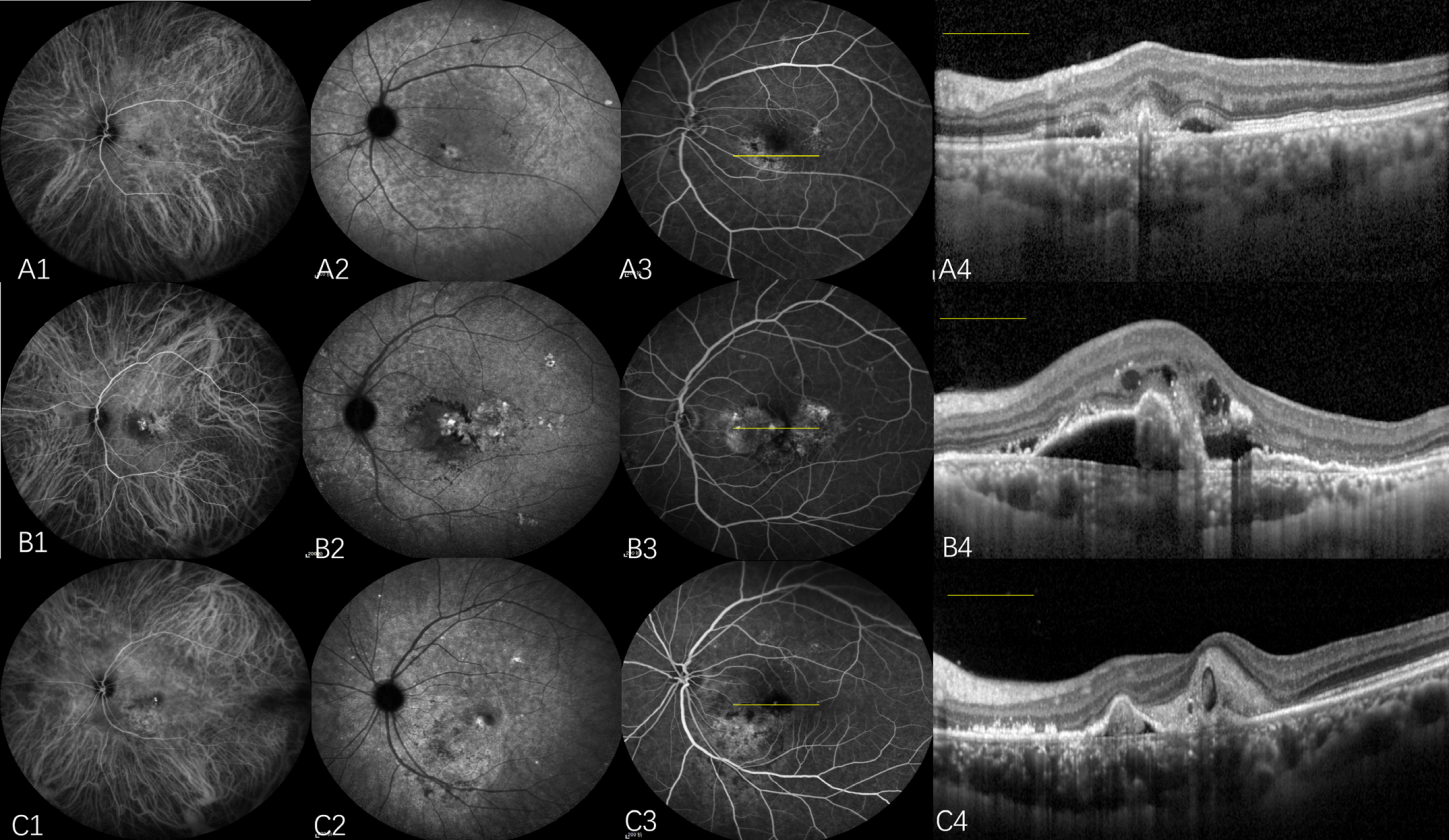
Multimodal imaging of ASHS-LIA in eyes with PCV. Three representative cases are shown, each demonstrating a different polypoidal lesion morphology: (**A**) a solitary polypoidal lesion, (**B**) grape-like polypoidal lesion clusters, and (**C**) clustered polypoidal lesions. (**A1**, **B1**, **C1**) Early-phase ICGA showed polypoidal lesions with no hypofluorescent spots. (**A2**, **B2**, **C2**) Late-phase ICGA showed hypofluorescent spots with partial confluence. (**A3**, **B3**, **C3**) Fundus fluorescence angiography (FFA) showed no corresponding abnormalities of ASHS-LIA. (**A4**, **B4**, **C4**) SD-OCTA B-scans (*yellow lines* in **A3**, **B3**, **C3**, respectively) clearly showed polypoidal lesions but no abnormalities correlating with the ASHS-LIA.

CVH was defined as patches of hyperfluorescence with indistinct contours during the middle phase (8–15 minutes) of ICGA following indocyanine green injection (Fig. 5).^23^ All included PCV eyes were categorized into the CVH group or non-CVH group. Age-related scattered hypofluorescent spots on late-phase indocyanine green angiography (ASHS-LIA) presented as hypofluorescent spots on late-phase ICGA (20–40 minutes after dye injection). Spots were distributed in the posterior pole, especially in the macular region, and regulated distribution could be confluent. There were no corresponding abnormalities on other forms of multimodal imaging, including color FP, FAF, FFA, and spectral-domain OCT (SD-OCT) (Fig. 6).^24^

The number and morphology of polypoidal lesions were analyzed based on ICGA. The total number of polyps was quantified, and their morphology was systematically classified based on early-phase images. To ensure clear and mutually exclusive categorization, a hierarchical approach was used, assigning each eye to one of the following three groups: (1) solitary, characterized by the presence of a single, isolated polypoidal lesion (Fig. 6A); (2) grape-like cluster, defined as a dense, tightly packed aggregation of multiple polypoidal lesions resembling a cluster of grapes and prioritized for classification due to its distinct morphology (Fig. 6B); and (3) clustered, which included any other grouping of two or more polyps that did not meet the specific criteria for a “grape-like cluster.” Lesions with a linear “string” configuration were also included in this group for the purpose of this analysis (Fig. 6C).^25^^–^^27^

All imaging results were evaluated by two masked, trained researchers (YZ and XC) to ensure the reliability and reproducibility of the study. If the two researchers could not reach a consensus, a third (FW) made the final evaluation. For the quantitative parameters, the mean value was calculated for analysis. The intraclass correlation coefficient (ICC) was calculated to evaluate the reproducibility.

### Statistical Analysis

Statistical analyses were performed using the SPSS Statistics 26.0 (IBM, Chicago, IL). ICCs were used to assess consistency in the measurement of choroidal parameters between the two image examiners. Descriptive statistics were reported as mean ± standard deviation (SD), median (interquartile range [IQR]), or as a number with percentage. Independent *t*-tests were applied to compare continuous variables between two groups. The Mann–Whitney *U* test and Pearson’s χ^2^ test were used to compare PCV eyes with and without CVH. The Wilcoxon signed rank test and Fisher's exact test were applied for comparisons of the affected eye and unaffected fellow eye. Statistical significance was defined as *P* < 0.05.

## Results

### Baseline Characteristics

As Table 1 shows, a total of 58 patients with treatment-naïve PCV in at least one eye were included in this study. The mean age was 64.3 ± 8.5 years, and the 38 patients were male (65.5%). Among the 58 patients, 50 were diagnosed with unilateral PCV, four were diagnosed with bilateral PCV, three had fellow eyes affected by typical AMD, and one had a fellow eye with pachychoroid neovasculopathy. For patients with bilateral PCV, we randomly selected one eye for analysis. Among the included 58 PCV eyes, CVH was observed in 32 eyes (54.2%). ASHS-LIA was observed in 31 eyes (53.4%). Twenty-seven PCV eyes (46.6%) had type 1 MNV, and 31 PCV eyes (53.4%) had mixed type 1 and type 2 MNV. The mean BCVA (logMAR) was 0.72 ± 0.58, The mean SFCT was 261.8 ± 70.6 µm. As for the lesion characteristics, the mean area of the BNN was 4.18 ± 4.15 mm^2^, and the mean number of polypoidal lesions was 2.69 ± 2.28 per eye. Regarding morphology, a clustered pattern was the most common (30/58, 51.7%), followed by solitary (18/58, 31.0%) and grape-like configurations (10/58, 17.2%).

**Table 1.** Demographics and Clinical Characteristics of Patients With PCV

Demographics and Clinical Features	Value
Patients (eyes), *n*	58
Age (y), mean ± SD	64.29 ± 8.49
Male, *n* (%)	38/58 (65.5)
BCVA (logMAR), mean ± SD	0.72 ± 0.58
ASHS-LIA, *n* (%)	31/58 (53.4)
CVH, *n* (%)	32/58 (54.2)
SFCT (µm), mean ± SD	261.83 ± 70.63
CVI (%), mean ± SD	42.33 ± 2.94
CVV/a (µm), mean ± SD	66.46 ± 22.41
CSV/a (µm), mean ± SD	86.24 ± 28.49
BNN area (mm^2^), mean ± SD	4.18 ± 4.15
Polypoidal lesions (*n*), mean ± SD	2.69 ± 2.28
Morphology of polypoidal lesions, *n* (%)	
Solitary	18/58 (31.0)
Clustered	30/58 (51.7)
Grape-like	10/58 (17.2)
MNV type, *n* (%)	
Type 1	27/58 (46.6)
Mixed type 1 and type2	31/58 (53.4)
Distribution of VVs, *n* (%)	
Symmetry	6/58 (10.3)
Asymmetry	52/58 (89.7)
Dominant VVs, *n* (%)	
One	25/58 (43.1)
Two	21/58 (36.2)
Three	6/58 (10.3)
Location of dominant VVs, *n* (%)	
SN	20/85 (22.0)
ST	29/85 (37.3)
IN	10/85 (6.8)
IT	26/85 (33.9)
Location of polypoidal lesions, *n* (%)	
SN	3/156 (2.0)
ST	72/156 (46.1)
IT	53/156 (34.0)
CWZ	28/156 (17.9)
Polypoidal lesions located in dominant VVs, *n* (%)	112/156 (71.8)

Of the 58 PCV eyes studied, 52 eyes (89.7%) had an asymmetric distribution of choroidal venous drainage routes, and the outflow tracts in the remaining six eyes (10.3%) were arranged symmetrically on WF-ICGA and en face OCTA images. Among the 52 eyes with asymmetric drainage patterns, one dominant VV was seen in 25 eyes, two dominant VVs were seen in 21 eyes, and three dominant VVs were seen in six eyes. Of these, the dominant VVs were more likely to be present in ST quadrants (29, 37.3%) and adhere to the ST–IT–SN–IN order for the descending number of dominant VVs (IT, 33.9%; SN, 22.0%; IN, 6.8%). A total of 156 polypoidal lesions were identified on ICGA and OCTA images. As with the location of dominant VVs, a majority of polypoidal lesions were located in the ST quadrant (72, 46.1%) and IT quadrant (53, 34.0%), and 28 (17.9%) were located at the horizontal watershed zone. Only three (2%) were located in SN quadrants, and no lesions were found in IN quadrants. Among the 52 eyes that had dominant VVs, 112 polypoidal lesions (71.8%) in 40 eyes (76.9%) were located within the quadrant of dominant VVs.

### Comparison of Demographic and Clinical Vascular Characteristics of PCV Eyes With and Without CVH

Table 2 shows a comparison of the demographic and clinical characteristics of PCV eyes with and without CVH. Among the 58 PCV eyes from 58 patients, 32 eyes had CVH (54.2%). There were no significant differences in patient characteristics (age, sex, and BCVA) or lesion features (BNN area, the number of polypoidal lesions, and morphology) between PCV eyes with and without CVH. There was a significant difference in SFCT, CVI, and CVV/a between the two groups (SFCT, *P* = 0.001; CVI, *P* = 0.004; CVV/a, *P* = 0.021). Our study also indicated that patients with CVH had less frequent ASHS-LIA than those without CVH (*P* = 0.001). For the MNV type of PCV, PCV eyes with CVH exhibited significantly more type 2 MNV (*P* = 0.01). WF-ICGA and WF-OCTA revealed a symmetric draining pattern in two eyes (6.3%) and an asymmetric fashion in 30 eyes (93.8%) among the CVH group, which did not reach statistical significance when compared to non-CVH eyes (*P* = 0.241). Meanwhile, the number and the location of dominant VVs did not differ significantly between the CVH and non-CVH groups (number, *P* = 0.592; location, *P* = 0.739).

**Table 2.** Comparisons of Demographic and Clinical Vascular Characteristics of PCV Eyes With or Without CVH

Parameter	CVH^+^ *(n* = 32)	CVH^–^ (*n* = 26)	*P*
Age (y), mean ± SD	62.38 ± 9.15	66.65 ± 7.07	0.055^a^
Male, *n* (%)	23 (71.9)	15 (57.7)	0.258^b^
BCVA (logMAR), mean ± SD	0.74 ± 0.579	0.69 ± 0.595	0.782^a^
ASHS-LIA, *n* (%)	11 (34.4)	20 (76.9)	0.001^b^
SFCT (µm), mean ± SD	289.75 ± 62.07	227.46 ± 66.11	0.001^a^
CVI (%), mean ± SD	43.28 ± 2.61	41.08 ± 2.90	0.004^a^
CVV/a (µm), mean ± SD	73.15 ± 22.08	58.92 ± 20.47	0.021^a^
CSV/a (µm), mean ± SD	92.92 ± 30.87	78.56 ± 23.85	0.070^a^
BNN area (mm^2^), mean ± SD	4.03 ± 3.71	4.35 ± 4.66	0.603^a^
Polypoidal lesions (*n*), mean ± SD	2.75 ± 2.68	2.62 ± 1.72	0.611^a^
Morphology of polypoidal lesions, *n* (%)			
Solitary	10/32 (31.3)	8/26 (30.8)	0.934^b^
Clustered	16/32 (50.0)	14/26 (53.8)	
Grape-like	6/32 (18.8)	4/26 (15.4)	
MNV type, *n* (%)			
Type 1	10/32 (31.3)	17/26 (65.4)	0.010^b^
Mixed type 1 and type2	22/32 (68.8)	9/26 (34.6)	
Distribution of VVs, *n* (%)			
Symmetry	2/32 (6.3)	4/26 (15.4)	0.241^c^
Asymmetry	30/32 (93.8)	22/26 (84.6)	
Dominant VVs, *n* (%), median (IQR)	1 (1∼2)	1 (1∼2)	0.592^d^
One	15/32 (46.9)	10/26 (38.5)	0.652^c^
Two	11/32 (34.4)	10/26 (38.5)	
Three	4/32 (12.5)	2/26 (7.7)	
Location of dominant VVs, *n* (%)			
SN	11/49 (22.4)	9/36 (25.0)	0.739^b^
ST	15/49 (30.6)	14/36 (38.9)	
IN	7/49 (14.3)	3/36 (8.3)	
IT	16/49 (32.7)	10/36 (27.8)	
Location of polypoidal lesions, *n* (%)			
ST	35/88 (39.8)	37/68 (54.4)	0.040^c^
IT	29/88 (33.0)	24/68 (35.3)	
SN	3/88 (3.4)	0/68 (/)	
CWZ	21/88 (23.9)	7/68 (10.3)	
Polypoidal lesions located in CWZ, *n* (%)			
CWZ	21/88 (23.9)	7/68 (10.3)	0.029^b^
Non-CWZ	67/88 (76.1)	61/68 (89.7)	
Polypoidal lesions located in dominant VV, *n* (%)	66/88 (75.0)	46/68 (67.6)	0.312^b^

a Independent samples *t*-test.

b Pearson's χ^2^ test.

c Fisher's exact test.

d Mann–Whitney *U* test.

**Table 3.** Comparisons of Choroidal Vein Drainage Pattern Between Affected Eyes and Unaffected Fellow Eyes

Parameters	Affected PCV Eyes (*n* = 50)	Unaffected Fellow Eyes (*n* = 50)	*P*
Distribution of VV, *n* (%)			
Symmetry	3/50 (6.0)	8/50 (16.0)	0.110^a^
Asymmetry	47/50 (94.0)	42/50 (84.0)	
Dominant VVs, *n* (%), median (IQR)	2 (1∼2)	1 (1∼2)	0.001^b^
One	21/50 (42.0)	28/50 (56.0)	0.013^a^
Two	20/50 (40.0)	14/50 (28.0)	
Three	6/50 (12.0)	—	
Location of dominant VVs, *n* (%)			
SN	16/79 (20.3)	22/56 (39.3)	0.108^a^
ST	29/79 (36.7)	14/56 (25.0)	
IN	9/79 (11.4)	5/56 (8.9)	
IT	25/79 (31.6)	15/56 (26.8)	
Temporal	54/79 (68.4)	29/56 (51.8)	0.039^c^
Nasal	25/79 (31.6)	27/56 (48.2)	

a Pearson's χ^2^ test.

b Fisher's exact test.

c Wilcoxon signed rank test.

A significant difference in polypoidal lesion distribution was observed between the CVH and non-CVH groups (*P* = 0.04). Specifically, the CVH group exhibited a significantly higher proportion of lesions located in the CWZ compared to the non-CVH group (23.9% vs. 10.3%; *P* = 0.029). The majority of polypoidal lesions were located in the ST quadrant in both groups (39.8% and 54.4%, respectively), and both groups adhered to the ST–IT–SN order for descending number of polypoidal lesions.

### Comparison Between Affected and Unaffected Eyes in Unilateral PCV

Fifty contralateral eyes derived from the 58 PCV eyes met the criteria for an unaffected fellow eye and were included as the control group for analysis. Table 3 shows the comparison of affected PCV eyes and unaffected fellow eyes from the 50 patients with unilateral PCV, affected eyes had a significantly larger number of dominant VVs than unaffected fellow eyes (*P* = 0.001). Six eyes (12.0%) exhibited three dominant VVs in the PCV eyes, but all unaffected fellow eyes exhibited only one or two dominant VVs. Also, compared to their unaffected peers, PCV eyes exhibited much more dominant VVs on the temporal side (68.4% vs. 51.8%; *P* = 0.039).

## Discussion

Using WF-ICGA and WF-OCTA, this study examined the spatial distribution of polypoidal lesions and dominant VVs in the CVH and non-CVH groups of PCV eyes and their unaffected fellow eyes. Our results show that PCV eyes exhibit a greater number of dominant VVs and a distinct distribution pattern compared to their fellow eyes, with a higher prevalence of temporal dominant VVs (*P* = 0.001 and *P* = 0.039, respectively). In alignment with the distribution of dominant VVs, the majority of polypoidal lesions (80.1%) were also localized in the temporal quadrant. Furthermore, 71.8% of polypoidal lesions were situated within the region of dominant VVs, demonstrating a strong correlation between lesion distribution and dominant VVs. Interestingly, although no significant differences were observed in the characteristics of dominant VVs between the CVH and non-CVH groups, the localization of polypoidal lesions exhibited notable variations (*P* = 0.04). Beyond the polypoidal lesions situated within the quadrant of dominant VVs, the remaining lesions were predominantly localized within CWZs in the CVH group compared to the non-CVH group (*P* = 0.029). This finding suggests that, although CVH was not found to directly change the distribution of dominant VVs, it may worsen the pathological microenvironment in CWZs, making these areas more vulnerable to vascular damage and lesion formation.

Our findings demonstrate the significance of dominant VVs in eyes with PCV. Previous results showed that PCD exhibited more imbalanced sizes of VV drainage territories compared to healthy individuals.^16^^,^^28^ However, research on the distribution of dominant VVs in PCV eyes remains limited. In the current investigation, the number of dominant VVs was significantly higher in individuals with unilateral PCV eyes than in eyes that were unaffected, and the dominant VVs were more frequently seen in the temporal side of PCV eyes. Furthermore, our study revealed that the majority of polypoidal lesions (71.8%) are located within the region of dominant VVs, a finding that aligns with the findings of a recently published study.^29^ In our study, we extended the analysis by investigating the quadrant distribution of dominant VVs and their spatial relationship with the quadrant locations of polypoidal lesions. Moreover, we conducted a comparative analysis between CVH and non-CVH eyes in PCV and investigated the distribution of dominant VVs between affected PCV eyes and their unaffected fellow eyes, enhancing our understanding of spatial characteristics of PCV pathogenesis, which is precisely the gap that our research addressed. In pathological states, increasing venous pressure can increase blood flow, resulting in increased wall shear stress.^30^ Due to the lesions mostly located in the temporal quadrant associated with temporal dominant VVs, we further propose that elevated shear stress in the temporal dominant VVs is a physiological factor that may lead to vascular endothelial injury, subsequently triggering abnormal vascular remodeling. Mori et al.^31^^,^^32^ demonstrated that half of normal eyes also had drainage asymmetry in their vortex vein and suggested that genetic susceptibility of PCV of stasis or overload in the vortex veins.^29^ In the current study, both affected and unaffected eyes exhibited a predominantly asymmetric choroidal vein outflow pattern. The observed binocular concordance of vortex vein engorgement in patients with unilateral PCV suggests that alterations in VV outflow may serve as a predisposing factor in the pathogenesis of this condition.^2^ Thus, we propose that the distribution and dominant VV characteristics are related to physiological change and may serve as a predisposing factor in the etiology in polypoidal lesion formation. Therefore, we consider that people with dominant VVs need to be alert to some predisposing factors such as smoking.^33^

Thirty-two PCV eyes (54.2%) in our study had CVH. However, the prevalence of CVH varies widely in eyes of PCV (9.8%–59.3%).^3^^,^^7^^,^^8^^,^^10^^,^^12^^,^^34^^–^^38^ This variation may arise from an evaluation of CVH based on qualitative image analysis. To our knowledge, our study is the first to systematically evaluate differences in choroidal vascularity in PCV eyes with or without CVH from WF-ICGA. As for the comparison between CVH group and non-CVH group, there were significant differences in ASHS-LIA, SFCT, CVI, CVV/a, and MNV type, which indicated that the CVH group and the non-CVH group may have had different underlying pathogenesis. Our previous study showed that 22.1% of the PCV eyes had type 2 MNV.^39^ In this study, we further evaluated the MNV type associated with CVH status. The results revealed that type 2 MNV was more prevalent in the CVH group. It is generally accepted that the presence of CVH indicates choroidal vein dilation, which may be brought on by increased choroidal pressure. We assume that, when the condition worsens, the compensatory capacity of the dilated choroidal vein becomes insufficient to counteract the choroidal hypertension, ultimately resulting in the protrusion of polypoidal lesions.

It is interesting to note that there was no significant difference in dominant VV characteristics between the CVH and non-CVH groups, but the localization of PCV lesions was significantly different. Other than the polypoidal lesions located within the dominant VV quadrant, the remaining polypoidal lesions were predominantly located within CWZs in the CVH group compared to the non-CVH group (*P* = 0.029). The CWZ, which represents the boundary between vascular territories supplied by distinct ciliary arteries, manifests as a transient hypofluorescent region that typically fades during the early phase of FA or ICGA.^21^^,^^22^ Previous studies have established CWZs as vulnerable regions of choroidal vascular perfusion, susceptible to pathological changes induced by ischemia and hypoxia.^40^ Additionally, research has demonstrated a topographic association between PCV lesion development and CWZs, implicating choroidal circulation dynamics in predisposing these regions to PCV growth.^41^^,^^42^ Our study has extended these insights by revealing that polypoidal lesions were preferentially located within CWZs in the CVH group, highlighting the critical role of CVH in the pathophysiology of PCV. This spatial predilection can be attributed to the inherent vulnerability of CWZs, characterized by limited collateral circulation and reduced blood perfusion, which predispose these regions to hypoxia and metabolic stress. Consequently, we hypothesize that CVH exacerbates this pathological microenvironment by inducing plasma leakage, thereby triggering a cascade of local hypoxia, VEGF upregulation, and inflammatory responses, ultimately culminating in vascular damage and polypoidal lesion formation. Importantly, CVH does not directly alter venous anatomy but amplifies the pre-existing pathological conditions within the CWZ, explaining why lesion localization differs between CVH and non-CVH groups despite similar venous distribution patterns (*P* = 0.029, *P* = 0.739, respectively). Although CVH does not directly alter VV distribution, it exacerbates the pathological microenvironment in CWZs, rendering these areas more susceptible to vascular damage. Together, these findings support the hypothesis that the formation and localization of PCV lesions are associated with congenital or acquired alterations in choroidal venous architecture, with elevated shear stress in temporal dominant VVs potentially contributing to lesion development.

The distinct anatomical and functional patterns of the choroidal venous architecture identified in our study hold significant clinical potential. Our findings suggest that dominant VV characteristics could serve as biomarkers for establishing personalized follow-up protocols for high-risk patients. Because venous asymmetry appears to be a constitutional factor, active surveillance of the fellow eye in unilateral PCV cases is also worth considering for early detection. Looking ahead, validating these anatomical markers in diverse ethnic groups is essential for developing robust risk stratification algorithms and advancing preventive care in PCV. A pragmatic consideration, however, is the translation of these findings into routine clinical practice, particularly in non-specialized centers.

Although our methodology relies on WF-ICGA and expert interpretation, which may not be universally available, its core logic can be simplified into a practical workflow. First, assess the location of a lesion relative to the dominant venous drainage. If the lesion is located within this dominant territory, anatomical factors such as the altered shear stress are probably the primary drivers, and management strategies might focus more on monitoring hemodynamic stability. Second, if the lesion lies outside this dominant venous territory, the clinician should then assess for the presence of CVH. A positive CVH status suggests that the development of the lesion is likely more significantly influenced by pathological changes within vulnerable CWZs, a process amplified by choroidal hyperpermeability. Therefore, a comprehensive management strategy should also consider addressing this choroidal vascular leakage. This simplified diagnostic framework will be the key for improving accessibility and guiding clinical decision-making.

This study had several weaknesses. First, the study was cross-sectional, without longitudinal follow-up to investigate the change of choroidal vascularity. Future studies will have to further investigate the longitudinal connections between choroidal vessels alterations and polypoidal lesion changes after treatment. Second, our sample size was relatively small. Third, comparative data from normal eyes were absent. Fourth, some assessments involved subjective judgment, even though we evaluated lesion locations using objective criteria.

In conclusion, the physiological choroidal venous drainage pattern and CVH-driven pathological changes may jointly influence PCV lesion formation and localization, highlighting the interplay between anatomical vulnerability and pathological processes in PCV pathogenesis. Additionally, the differential distribution of dominant VVs highlights the significance of anatomical factors in PCV development. These findings offer a new perspective for comprehending PCV mechanisms.
